# Electrochemical Immunoassay Platform for Human Monkeypox Virus Detection

**DOI:** 10.1021/acs.analchem.3c05182

**Published:** 2024-05-10

**Authors:** Göksu Can, Benay Perk, Burak Ekrem Çitil, Yudum Tepeli Büyüksünetçi, Ülkü Anık

**Affiliations:** † Faculty of Science, Chemistry Department, Mugla Sitki Kocman University, Kotekli, Mugla 48000, Turkey; ‡ Faculty of Medicine, Department of Medical Microbiology, Mugla Sitki Kocman University, Kotekli-Mugla 48000, Turkey; § Research Laboratory Center, Mugla Sitki Kocman University, Kotekli, Mugla 48000, Turkey; ∥ Sensors, Biosensors and Nano-diagnostic Systems Laboratory, Research Laboratory Center, Mugla Sitki Kocman University, Kotekli, Mugla 48000, Turkey

## Abstract

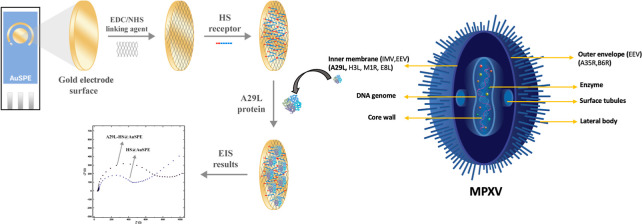

In this study, we reported a selective impedimetric biosensor for the detection of A29 which is the target protein of the monkeypox virus (MPXV). The working principle of the biosensor relies on the interaction mechanism between A29, which is an internal membrane protein of MPXV, and the heparan sulfate receptor. For this purpose, after immobilizing heparan sulfate onto the gold screen-printed electrode surface, its interaction with A29 protein was monitored using electrochemical impedance spectroscopy. After the optimization of experimental parameters, the analytical characteristics of the developed MPVX immunosensor were examined. The developed immunosensor exhibited a linear detection range between 2.0 and 50 ng mL^–1^, with a detection limit of 2.08 ng mL^–1^ and a quantification limit of 6.28 ng mL^–1^. Furthermore, a relative standard deviation value of 2.82% was determined for 25 ng mL^–1^. Apart from that, sample application studies were also performed with the standard addition of A29 protein to 1:10 diluted real serum samples that were taken from healthy individuals, and very good recovery values were obtained.

## Introduction

Monkeypox can simply be described as a closely relevant pox disease in monkeys that is caused by the monkeypox virus (MPXV). MPXV is in the *Poxviridae* family and belongs to genus *Orthopoxvirus*.^1−4^ Multiple neurological and physiological symptoms such as fatigue, headache, weakness, altered consciousness, nausea, and vomiting are reported in patients infected with this virus.^5^ Monkeypox was used to define as an endemic African disease that causes not so much serious problems globally. Unfortunately, an increasing number of monkeypox cases have been reported in disparate regions of the world in early May 2022,^6^ and since it has been seen in more than 28 countries apart from African countries, recently it is declared as global public health emergency by the World Health Organization (WHO).^1,7,8^ In this manner, it seems possible to have a monkeypox pandemic in the future considering the presence of asymptomatic human beings and the lack of massive MPXV testing.^1^ For this reason, rapid, effective, and economical detection tools for MPXV identifications are urgently needed.

Until now, there are some monkeypox diagnostic methods that have been used for diagnosing the infections. Some of these techniques are polymerase chain reaction,^9,10^ virus culture-based diagnosis,^11^ protein Cas-based diagnosis,^12,13^ loop-mediated isothermal amplification,^14,15^ enzyme-linked immunosorbent assay,^16,17^ and recombinase polymerase amplification assay.^1,9,18^ Considering the point-of-care (POC) nature of rapid tests, antibody-based immunoassays seem to be stronger diagnostic tool candidates for MPXV detection. However, due to the antigen context of *Orthopoxvirus* genomes, most human samples become cross-reactive, and this situation makes it impossible to differentiate different kinds of *Orthopoxvirus* viruses.^19−21^

A protein that can be specific to MPXV has been reported by Hughes et al. in a study that was published in 2014.^19^ In that study, A29 protein was found to be reacted with MPXV-specific monoclonal antibody (mAb 69-126-3-7).^22^ Also, it was reported that an epitope of this protein has been identified as having a single amino acid difference which allows the usage of A29 protein for the design of monkeypox-specific serological assays.^19^ Apart from that, the binding of this protein with heparin was tested, and it was observed that monkeypox A29 protein bound to heparin with similar affinity to that of VACV A27 protein.^19^

Based on their practicality, accuracy, and specificity, biosensors offer great advantages in terms of POC systems. Because of the recent SARS-CoV-2 pandemic, many rapid tests that were based on biosensors have been fabricated.^23,24^ Electrochemical biosensors are one of the most popular biosensors because of their practicality, accuracy, and low cost. Among them, especially impedimetric biosensors have been vastly produced and used because of their versatility. In accordance with this, herein, we propose an impedimetric biosensor for MPXV detection for the first time. In this biosensor, for diagnosing monkeypox disease, the infection mechanism of MPVX was followed. For this purpose, after immobilization of heparan sulfate (HS) on the electrode surface, the interaction between A29 protein and HS was followed via electrochemical impedance spectroscopy (EIS). Here, the usage of HS mimicked the healthy cells, while A29 protein belonged to the MPVX structure. After optimization of the working conditions, the standard addition responses of A29 to real serum samples were recorded.

## Experimental Section

### Materials

Potassium dihydrogen phosphate (KH_2_PO_4_) and sodium hydroxide (NaOH) were purchased from Merck. Urea, Tris–HCl, K_3_Fe(CN)_6_, K_4_Fe(CN)_6_, *N*-hydroxysuccinimide (NHS), and *N*-(3-(dimethylamino)propyl-)-*N*′-ethylcarbodiimide (EDC) were obtained from Sigma-Aldrich. H1N1 influenza virus was obtained from HyTest. KMP-11 antigen was available from Creative Diagnostics. HS was provided from Origene. Recombinant A27 protein was purchased from Abcam, and recombinant MPXV protein A29 was provided from Sino Biological Inc. AuSPE was purchased from Dropsens. All reagents and chemicals employed were of analytical purity and utilized in their acquired state from corporate sources. All the other solutions were prepared with ultrapure water from Bluaqua Kapelle series ultrapure water systems.

### Instrumentation

Throughout the experiments, a commercial gold screen-printed electrode (AuSPE) immunosensor transducer was used. AuSPE included a gold working electrode (diameter = 4 mm), a platin counter electrode, and a silver reference electrode on a single platform. Additionally, EIS and cyclic voltammetry (CV) were performed using a μ-AUTOLAB potentiostat equipped with NOVA 1.10 software and an FRA-2 module.

Atomic force microscopy (AFM) analyses were done with the tapping mode technique, 15–29 kHz resonant frequencies, and all images were acquired in air at ambient conditions. For scanning electron microscopy (SEM) analysis, samples were coated with a gold/palladium (Au/Pd) target plate with a coating thickness of 9 nm using a Leica EM ACE600 sample coater. Imaging of the coated samples was carried out with a chamber pressure of 1.00–3 Pa and a resolution of 1 nm at 1 kV.

A pH meter made by Thermo Detection Corporation was used to check the buffer solutions’ pH levels. A Velp Scientifica vortex mixer was used to homogenize the solutions thoroughly, and a BIOSAN environmental shaker, incubator ES-20 was used for incubation.

### Fabrication and Electrochemical Detection Assay of Impedimetric Monkeypox Immunoassay

**Scheme 1:**
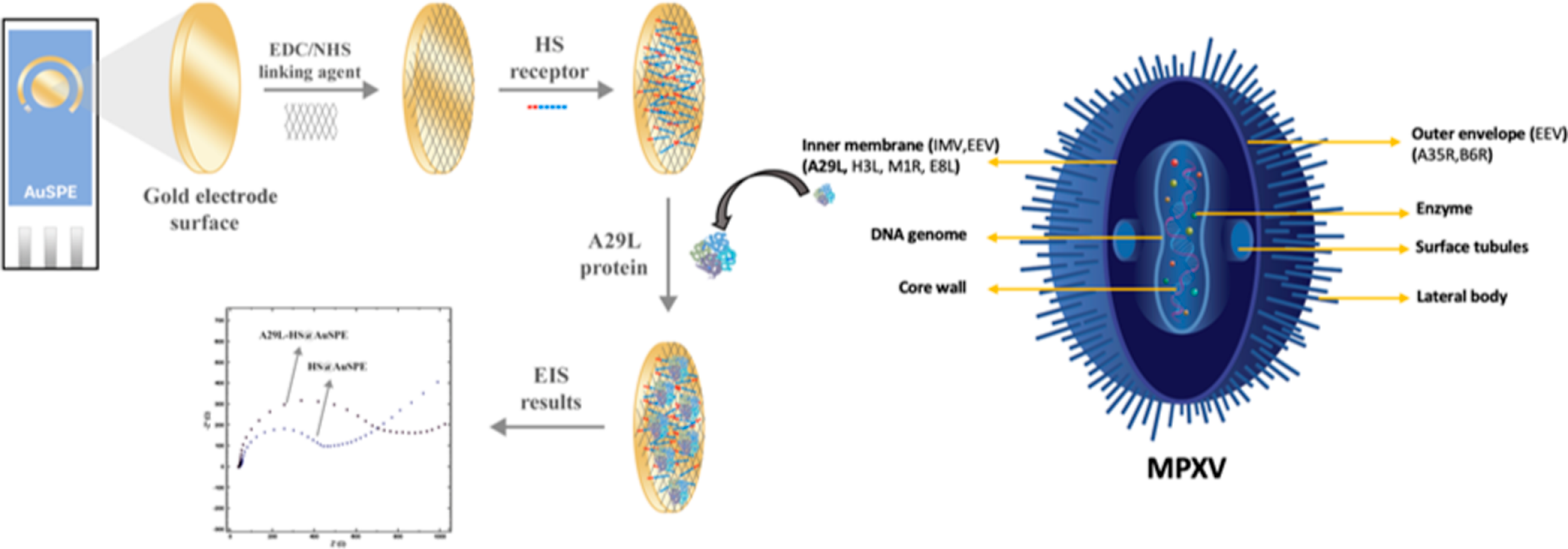
Schematic Representation of the Fabrication of the Developed MPXV Immunosensor

For the preparation of impedimetric monkeypox assay, first, 10 μL of 100 mM EDC and 10 μL of 150 mM NHS were dropped onto the AuSPE surface and dried at 25 °C for 10 min. Then, 50 μL of HS (2 ng mL^–1^ in pH: 8.0 Tris–HCl buffer) was immobilized onto the electrode surface at 25 °C for 30 min. Eventually, 10 μL of 25 ng mL^–1^ A29 protein was dropped onto HS@AuSPE and left at 4 °C for 30 min in order to complete the HS and A29 protein incubation procedure. After the incubation procedure, the electrode surface was rinsed with 1.5 mL of 0.1 M phosphate buffer solution (buffer solution was prepared by adjusting the solution prepared with KH_2_PO_4_ to pH 7.4 with 3 M NaOH), and the resistance value of A29-HS@AuSPE was investigated by EIS (Scheme 1).

The EIS working frequency in all trials was between 0.1 Hz and 100 kHz at a potential of 0.1 V. The conductance change of modified and unmodified AuSPE was measured in the presence of 70 μL of 5 mM [Fe(CN)_6_]^3–/4–^ redox probe couple, and 1.5 mL of phosphate buffer solution (pH: 7, 4) was used for the removal of impurities in between all the steps.

The working principle of the developed electrochemical biosensor was based on the changes in resistance measurements as a result of immobilization of HS and A29 protein on AuSPE. The main reaction used in this electrochemical biosensor was the specific HS–A29 protein interaction that was followed by an increment in the resistance value. Meanwhile, all the measurements were performed in triplicate, and the results with calculated standard deviations were presented with error bars.

Also for electrochemical characterization, the CV method was applied with the working potential range of 0.8–1.0 V and a scan rate of 0.1 mV s^–1^. The measurements were performed in the presence of 70 μL of a 5 mM [Fe(CN)_6_]^3–/4–^ redox probe in 0.1 M phosphate buffer solution (pH: 7.4).

### Selectivity Study

A review of the literature revealed that the biological agents that could interact with the HS receptor are H1N1, A27L protein, and leishmania parasite antigen KMP11.^23−25^ Consequently, these three biological agents were used in 1:1 and 1:2 ratios in the presence of 25 ng mL^–1^ A29 protein for the interference studies.

### Real Sample Application

Sample experiments were performed by adding an appropriate amount of A29 protein to 1:10 diluted serum samples that were obtained from healthy individuals. 7.5, 12.5, 25, and 50 ng mL^–1^ A29 protein were added to the samples, and measurements were performed in three replicates. The results obtained in triplicate were presented as a calibration graph.

## Results and Discussion

### Electrochemical Characterization of the Monkeypox Virus Biosensor

**Figure 1:**
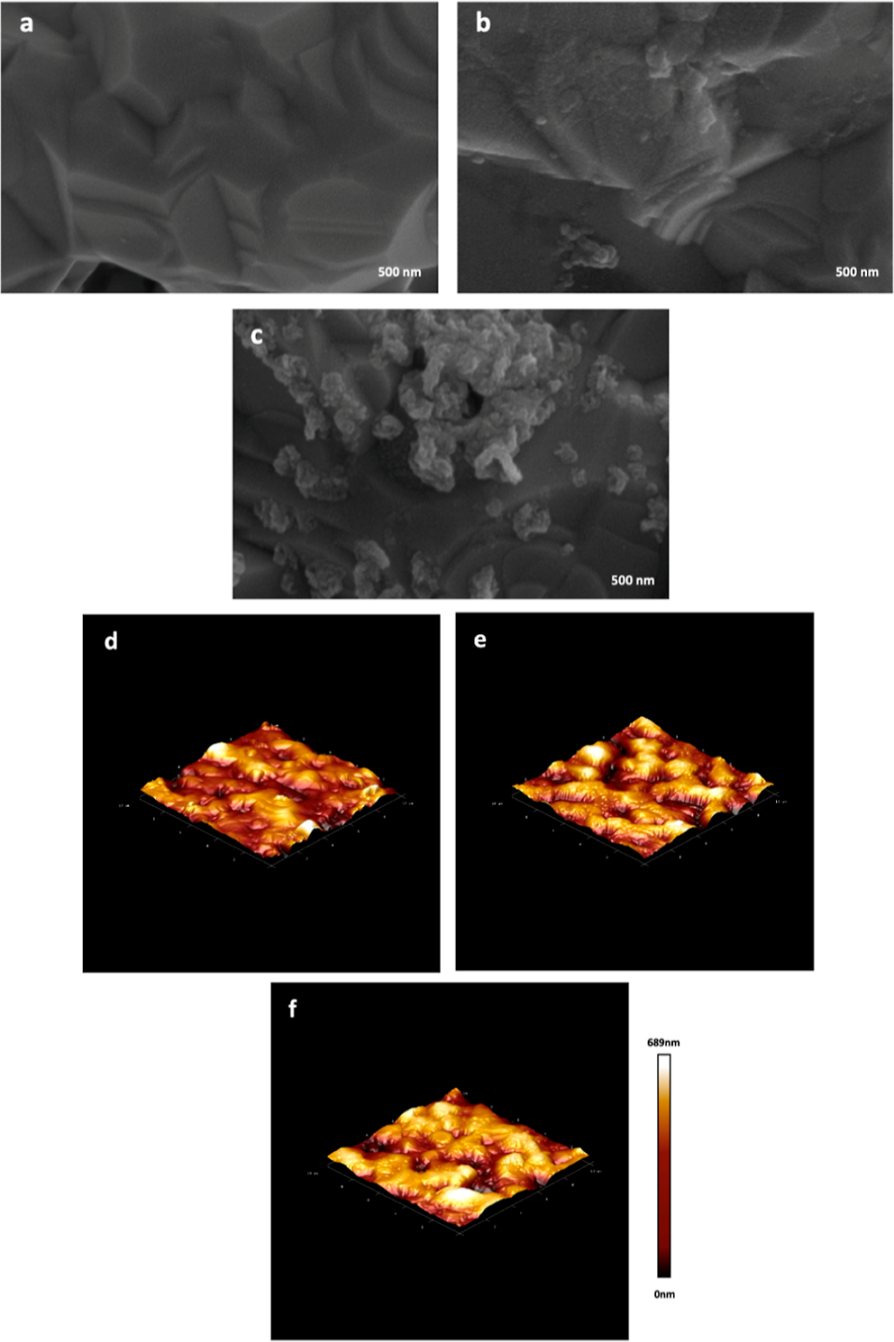
SEM (a–c) and AFM (d–f) images of bare AuSPE, HS@AuSPE, and A29@HS@AuSPE, respectively.

For the characterization of the developed biosensor, SEM and AFM analyses were performed (Figure 1). Figure 1a–c shows the SEM images of bare AuSPE, HS@AuSPE, and A29 protein immobilized on HS@AuSPE surfaces, respectively. As can be seen from the figure, no structure is visible on bare AuSPE (Figure 1a). On the other hand, after each immobilization step, round spheres that were denoted as immobilized receptors and proteins on the AuSPE surface were seen on SEM images (Figure 1b,c). Figure 1d–f shows the 3D AFM images of bare AuSPE, HS@AuSPE, and A29 protein immobilized on HS@AuSPE, respectively. From the images that belonged to each electrode preparation step, spheres in the form of small grains and aggregation in the form of hills on the electrode surface were observed. Especially for A29 immobilization, a distinct protein coating can be seen in AFM imaging as 3D hills and dotted shapes (Figure 1f). From these images, it was concluded that aimed immobilization steps were performed successfully.

**Figure 2:**
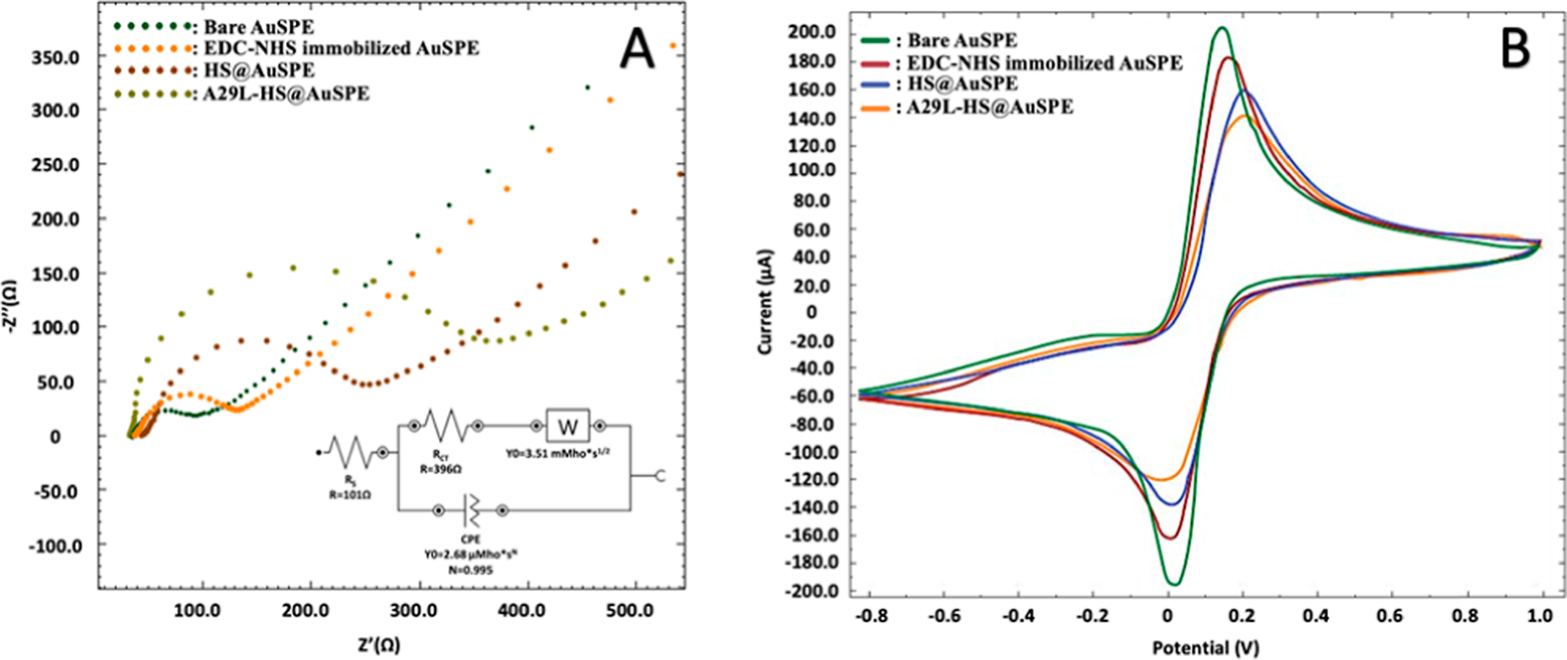
Nyquist diagrams of resistance measurements and their equivalent circuit (A) and CV voltammogram (B) of each preparation stage of the developed electrochemical MPXV biosensor. The frequency was from 0.1 Hz to 100 kHz at 0.1 V potential for EIS, and the working potential range for CV was between −0.8 and 1.0 V with 0.1 mV s^–1^ scan rate. The measurements were taken in the presence of 70 μL of a 5 mM [Fe(CN)_6_]^3–/4–^ redox probe in 0.1 M phosphate buffer solution (pH: 7.4). *R*_S_ is the resistance of the electrolyte solution. For the equivalent circuit, *R*_CT_ is the electron-transfer resistance of the electrode/electrolyte interface, CPE is the constant phase element, and *W* is the Warburg impedance for semi-infinite diffusion.

Also, EIS and CV methods were used for the electrochemical characterization of the developed immunoassay.^25^ As can be seen in Figure 2A, bare AuSPE exhibits the smallest Nyquist curve due to the conductive gold working electrode surface. After EDC–NHS immobilization, an increase in the Nyquist curve was observed compared to that of bare AuSPE as expected. This indicates the coverage of the Au surface with EDC–NHS. In the next step, upon the attachment of HS receptor on the electrode surface, an increase in resistance was observed as this hindered the electron-exchange kinetics in the active electrode surface area. Conclusively, A29 protein, which binds specifically to the HS receptor, was immobilized on the AuSPE surface, and resistance-dependent increases were observed in the Nyquist curves as the AuSPE surface became more coated at each step (Figure 2A). On the contrary, in CV voltammograms, bare AuSPE has the highest current value because of the fastest electrode transfer on its surface (Figure 2B). Also, the current value decreases as new immobilization layers were formed on the electrode surface because of the blockage of electron transfer (Figure 2B). These results are in accordance with the successful coverage of electrode surface with each immobilization step.

### Optimization of Experimental Parameters

The developed electrochemical MPXV biosensor was optimized for obtaining the best accuracy by varying the HS amounts and incubation times of HS and A29 protein. The optimizations were performed in the presence of 70 μL of 5 mM [Fe(CN)_6_]^3–/4–^ redox probe at a constant potential of 0.1 V by EIS method in three replicates. The operating conditions were chosen to be physiological human pH and body temperature, so all experiments were performed at pH 7.4 and 37 °C except the A29 and HS incubation process.

### Effect of HS Amount on an Electrochemical MPXV Biosensor

**Figure 3:**
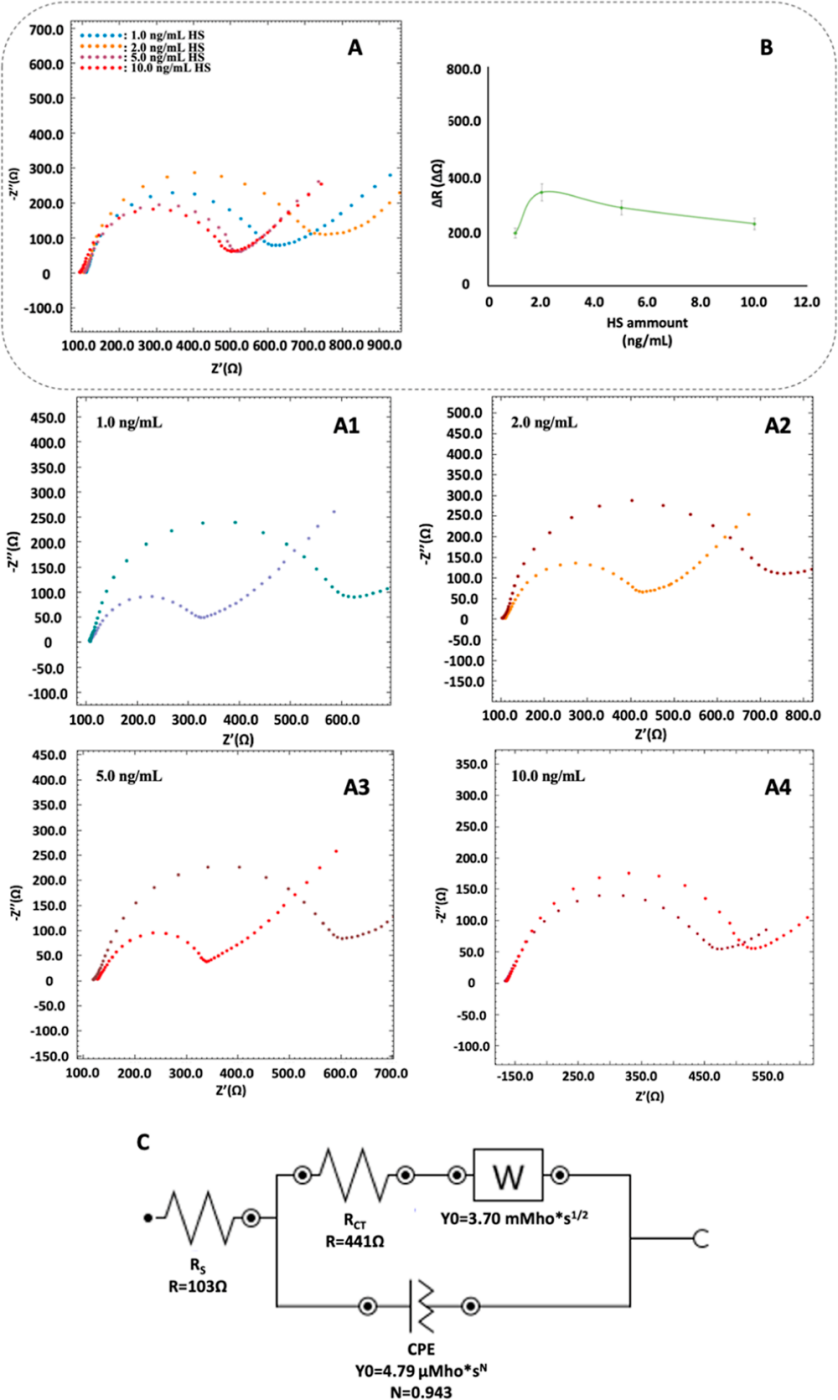
Nyquist diagrams (A) and impedance difference excel plot (B) of HS amount (1.0, 2.0, 5.0, and 10.0 ng mL^–1^) optimization in terms of resistance difference. Closer demonstrations of impedance differences for different HS amounts (A1–A4). Representation of the equivalent circuit for the optimum condition of 2 ng/mL HS. For the equivalent circuit, *R*_S_ is the resistance of the electrolyte solution, *R*_CT_ is the electron-transfer resistance of the electrode/electrolyte interface, CPE is the constant phase element, and *W* is the Warburg impedance for semi-infinite diffusion (C). Δ*R*: Resistance difference. All other experimental conditions as in Figure 2

To optimize the amount of HS in the developed electrochemical biosensor, immunoassays containing 50 μL of 2, 5, 10, and 15 ng mL^–1^ HS were prepared. Their responses to A29 protein were investigated via EIS in the presence of 25 ng mL^–1^ A29 protein, 100 mM EDC, and 150 mM NHS. Among the biosensors prepared using different amounts of HS, the highest resistance increase was observed at 2 ng mL^–1^, demonstrating the optimum coverage of electrode in terms of HS receptor (Figure 3A) For this reason, the optimum amount of HS was selected as 2 ng mL^–1^ (Figure 3A2).

### Optimization of HS-A29 Incubation Time

**Figure 4:**
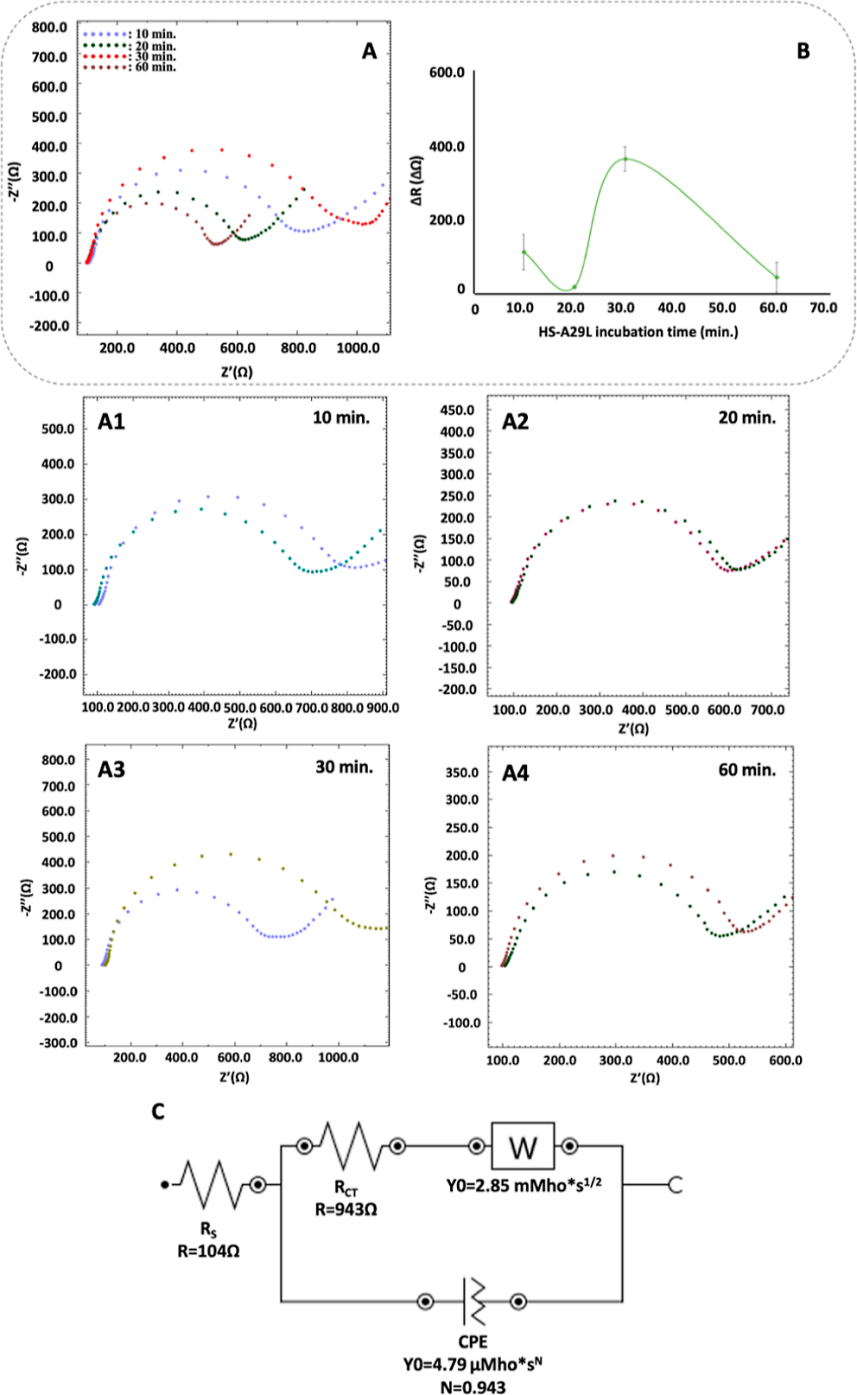
Nyquist diagrams (A) and impedance difference excel plot (B) of HS and A29 protein incubation time optimization studies (10, 20, 30, and 60 min). Closer demonstrations of differences in EIS diagrams for HS and A29 protein interaction (A1–A4). Representation of the equivalent circuit for the optimum condition of 30 min incubation time. For the equivalent circuit, RS is the resistance of the electrolyte solution, RCT is the electron-transfer resistance of the electrode/electrolyte interface, CPE is the constant phase element, and *W* is the Warburg impedance for semi-infinite diffusion (C). Δ*R*: Resistance difference. All other experimental conditions as in Figure 2

The fact that the developed immunosensor has fast response time is an important parameter in terms of practicality.^1^ On the other hand, effective interaction between the analyte and receptor has an influence on the accuracy of the developed system. Therefore, the duration of interactions between the HS and A29 protein was optimized. For this purpose, 10, 20, 30, and 60 min incubation times were applied under the optimum working conditions at a temperature of 4 °C.^26^ As a result, it was observed that the resistance values had a gradual increase until the 30th minute and then a decrease at the 60th minute. Following this finding, 30 min was selected as the optimum interaction time and used in subsequent studies (Figure 4A3).

### Analytical Characteristics

**Figure 5:**
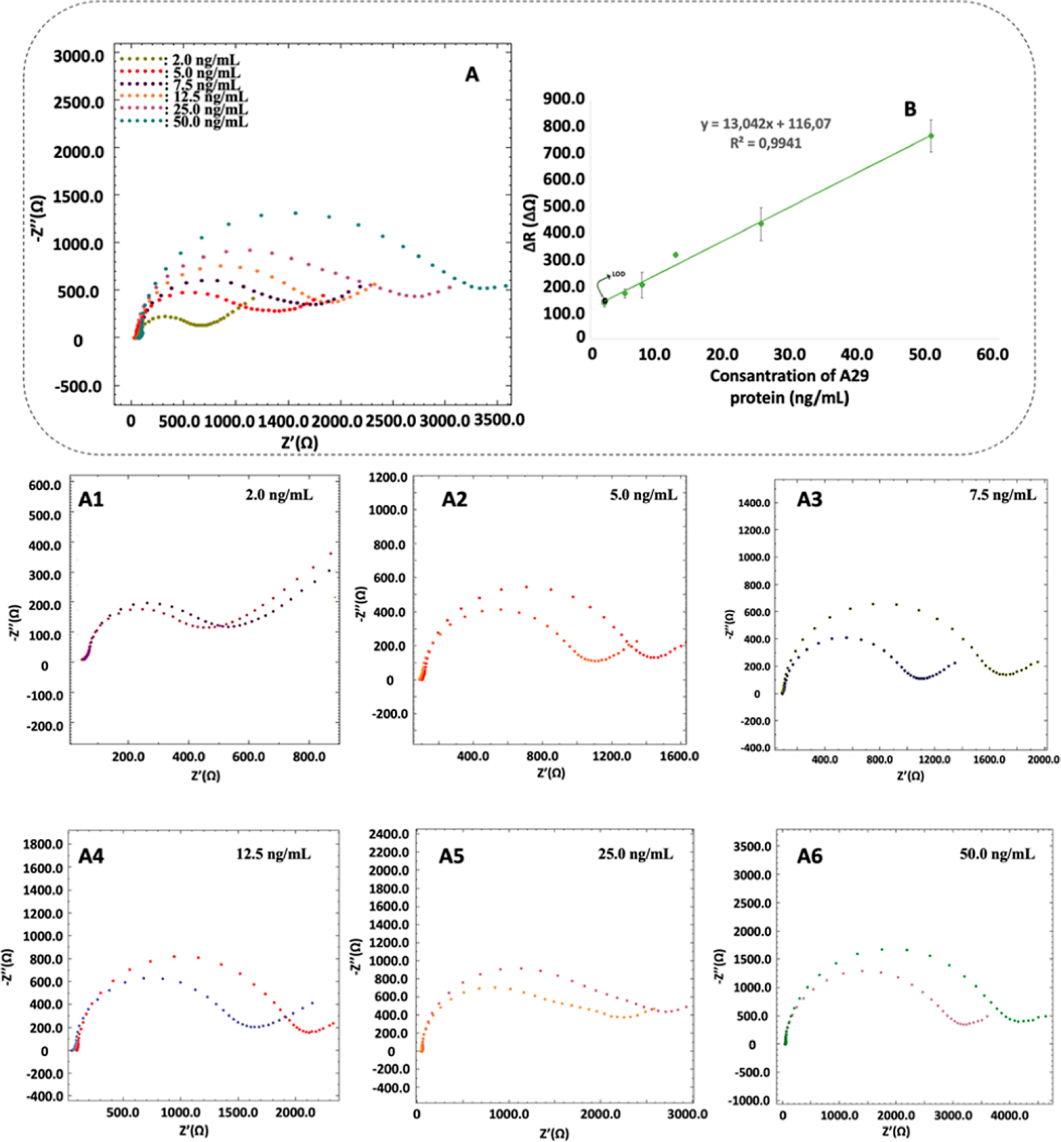
Nyquist diagrams (A) and LOD value included calibration curve (B) of the developed electrochemical MPXV biosensor with increasing A29 protein concentrations (2.0, 5.0, 7.5, 12.5, 25.0, and 50.0 ng mL^–1^). Closer demonstration of impedimetric responses for various A29 concentrations (2.0–50 ng mL^–1)^ (A1–A6). Δ*R*: Resistance difference. All other experimental conditions are listed in Figure 2

After the optimization of the experimental parameters, linear response range of the developed immunosensor was investigated using A29 protein solutions at concentrations of 2.0, 5.0, 7.5, 12.5, 25.0, and 50.0 ng mL^–1^. As can be seen in Figure 5B, there is a linear response range between 2 and 50 ng mL^–1^ concentrations with the equation of *y* = 13.041*x* + 116.07 (*R*^2^ = 0.9934). Limit of detection (LOD) and limit of quantification (LOQ) values can be used to evaluate the sensitivity of the developed biosensor. For this reason, LOD (defined as 3 s/m; s: 8.24, which is the standard deviation of the blank, and m: 13.042, which is slope of the calibration curve) and LOQ (defined as 10 s/m) values were calculated as 2.08 and 6.28 ng mL^–1^, respectively. In addition, the relative standard deviation value for 25 ng mL^–1^ A29 protein was calculated as 2.82% (*n* = 3).

The analytical characteristics of the developed MPVX immunosensor were compared with CRISPR methods as demonstrated in Table 1A. Though CRISPR methods had lower detection limits with wider linear ranges, the A29-based MPVX immunosensor is more practical and faster than these methods.

**Table 1:** Comparison of Various Methods Reported for MPXV Detection

technique	target	linear range	LOD	reference
CRISPR	F3L gene	1 × 10^1^ to 1 × 10^4^ copies μL^–1^	1.0 × 10^1^ copies μL^–1^	(12)
CRISPR	DNA	1 × 10^0^ to 1 × 10^6^ copies μL^–1^	13.5 copies μL^–1^	(31)
EIS	A29L	2 × 10^–9^ to 50 × 10^–9^ g mL^–1^	2.08 × 10^–9^ g mL^–1^	this study

### Interference Study

**Figure 6:**
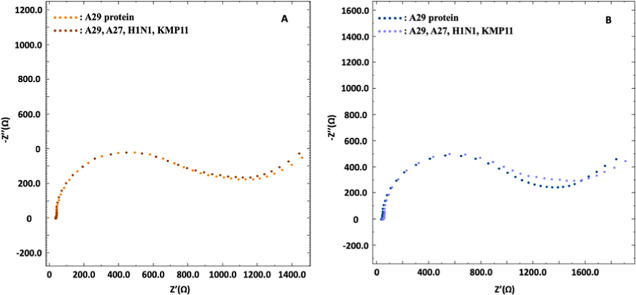
Nyquist diagrams of 1:1 (A) and 1:2 (B) ratios of interferent cocktails for interference studies. All other experimental conditions as in Figure 2

Interference experiments were performed under optimized conditions in the presence of 25 ng mL^–1^ A29 protein where the concentration of cocktail mixtures of possible interferents was in the ratio of 1:1 and 1:2 related to the concentration of A29. Following the information in the literature, A27, KMP11, and H1N1 were selected as possible interferents.^27−29^ Using the results obtained from the EIS measurements of 1:1 and 1:2 cocktail mixtures, recovery values were calculated as 98.2 and 104%, respectively (*n* = 3). These recovery values clearly demonstrate the usage of the developed immunosensor in the presence of the above interferents (Figure 6).

### Sample Application

**Figure 7:**
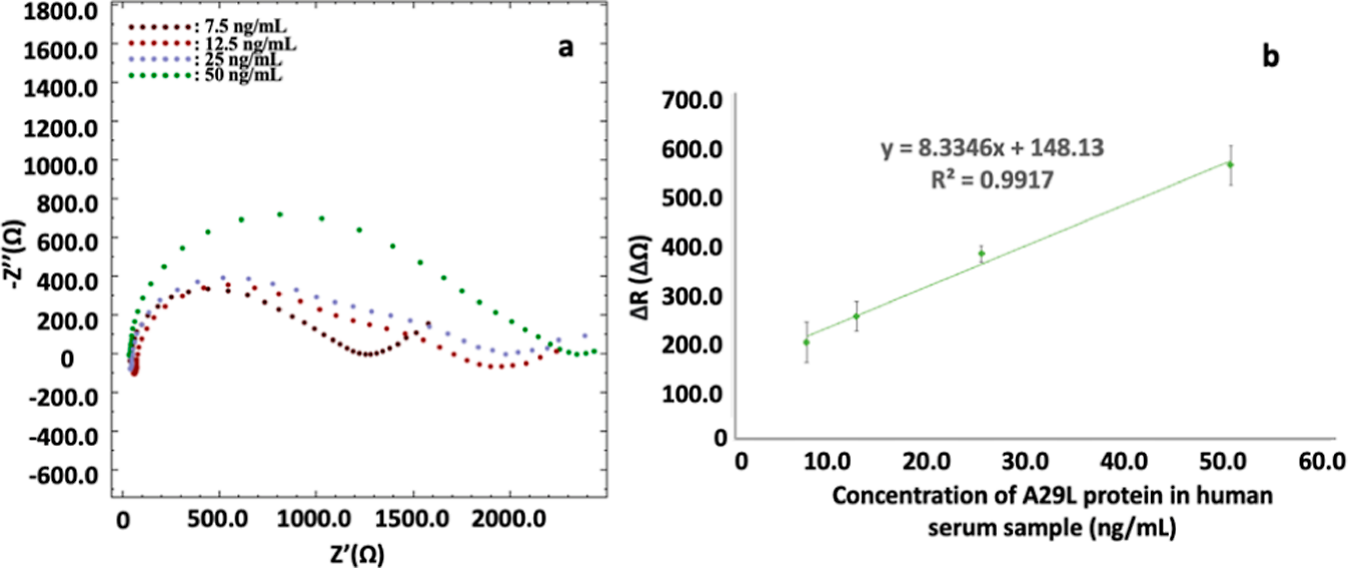
Sample application studies of the developed monkeypox electrochemical immunosensor. Nyquist diagrams (a) and excel graph (b) for standard addition of 7.5, 12.5, 25, and 50 ng mL^–1^ A29 protein to 1:10 diluted human serum samples. ΔR: Resistance difference. Working conditions are as in Figure 2

Sample application studies were performed by using real serum samples via a standard addition technique. For this purpose, A29 protein (at concentrations of 7.5, 12.5, 25, and 50 ng mL^–1^) prepared in human serum samples diluted in the ration of 1:10 was used in the fabrication of an electrochemical immunosensor. The interaction of the prepared electrochemical immunosensor with HS under optimum conditions was obtained by EIS, and the experiments were carried out in three replicates. As can be seen in Figure 7a, an increase in resistance values was observed as the added A29 concentration was increased.

## Conclusions

The importance of biosensors as diagnostic tools has been well understood during the recent COVID-19 pandemic. The nature of these systems offers many valuable opportunities for the construction of effective POC systems. From this point of view, by using monkeypox-specific protein A29, herein we manage to develop an effective and selective electrochemical monkeypox immunosensor. Because of A29 –HS interactions and electrochemical nature, the resulting immunosensor possesses specificity and hence practicality, eliminating the serological cross reactions which many *Orthopox* viruses openly have. Considering the health emergency alert for MPXV, we believe that our system has the potential and could be converted into a facile POC diagnostic tool for the future.

## Notes

This study is a patent pending study with application number 2023/004290.
